# The evolutionary history of protein fold families and proteomes confirms that the archaeal ancestor is more ancient than the ancestors of other superkingdoms

**DOI:** 10.1186/1471-2148-12-13

**Published:** 2012-01-27

**Authors:** Kyung Mo Kim, Gustavo Caetano-Anollés

**Affiliations:** 1 Evolutionary Bioinformatics Laboratory, Department of Crop Science, University of Illinois, Urbana, IL 61801, USA; 2 Korean Bioinformation Center, Korea Research Institute of Bioscience and Biotechnology, 111 Gwahangno, Yuseong-gu, Daejeon 305-806, Korea

## Abstract

**Background:**

The entire evolutionary history of life can be studied using myriad sequences generated by genomic research. This includes the appearance of the first cells and of superkingdoms Archaea, Bacteria, and Eukarya. However, the use of molecular sequence information for deep phylogenetic analyses is limited by mutational saturation, differential evolutionary rates, lack of sequence site independence, and other biological and technical constraints. In contrast, protein structures are evolutionary modules that are highly conserved and diverse enough to enable deep historical exploration.

**Results:**

Here we build phylogenies that describe the evolution of proteins and proteomes. These phylogenetic trees are derived from a genomic census of protein domains defined at the fold family (FF) level of structural classification. Phylogenomic trees of FF structures were reconstructed from genomic abundance levels of 2,397 FFs in 420 proteomes of free-living organisms. These trees defined timelines of domain appearance, with time spanning from the origin of proteins to the present. Timelines are divided into five different evolutionary phases according to patterns of sharing of FFs among superkingdoms: (1) a primordial protein world, (2) reductive evolution and the rise of Archaea, (3) the rise of Bacteria from the common ancestor of Bacteria and Eukarya and early development of the three superkingdoms, (4) the rise of Eukarya and widespread organismal diversification, and (5) eukaryal diversification. The relative ancestry of the FFs shows that reductive evolution by domain loss is dominant in the first three phases and is responsible for both the diversification of life from a universal cellular ancestor and the appearance of superkingdoms. On the other hand, domain gains are predominant in the last two phases and are responsible for organismal diversification, especially in Bacteria and Eukarya.

**Conclusions:**

The evolution of functions that are associated with corresponding FFs along the timeline reveals that primordial metabolic domains evolved earlier than informational domains involved in translation and transcription, supporting the metabolism-first hypothesis rather than the RNA world scenario. In addition, phylogenomic trees of proteomes reconstructed from FFs appearing in each of the five phases of the protein world show that trees reconstructed from ancient domain structures were consistently rooted in archaeal lineages, supporting the proposal that the archaeal ancestor is more ancient than the ancestors of other superkingdoms.

## Background

Since Darwin established the general principles of natural selection in 1859 [1] and Kimura proposed the neutral theory in the late 1960s [2], most evolutionary studies have focused on individual gene sequences. Molecular sequences of nucleic acids or proteins clarify evolutionary relationships among closely related species defined for example at the genus or family levels. However, their information is not sufficient to survey deep phylogenetic information. For example, deep branches at the base of the group of ribosome-containing organisms that define the three cellular superkingdoms of life, Archaea, Bacteria, and Eukarya, are not resolved in a tree of organisms based on ribosomal RNA (rRNA) sequences [3]. These trees describe the history of only one of the many protein and RNA molecules that make up the ribosomal ensemble but are nevertheless regarded as reference for species phylogeny. However, the recent revolution in nucleic acid sequencing driven by shotgun and high-throughput technologies (e.g., pyrosequencing, Illumina, SOLiD, etc) has led to the rapid generation of myriad genomic sequences across the three superkingdoms and viruses. It has been expected that genomic sequence information will be sufficient to elucidate phylogenetic relationships that were not resolved before. In this regard, phylogenetic approaches based on genome sequences (e.g., sequence concatenation) and the genomic content of genes has been successfully used to build phylogenies at various taxonomic levels, including trees of organisms [4,5]. However, these approaches are problematic since only a limited proportion of entire gene families in the studied genomes are orthologous and available for tree reconstruction [6]. Furthermore, molecular sequences suffer from the effects of a number of important constraints, including saturation by rapid mutational change (substitutions and indels), non-orthologous gene replacement, differential rates of evolution in lineages, horizontal gene transfer, lineage sorting by sequence polymorphisms, and paralogous relationships by gene or genome duplication [7-9]. By definition, sequence sites are not independent from each other because of molecular structure, thus violating the phylogenetic character independence requirement of phylogenetic analysis. Furthermore, a substantial number of protein-encoding genes are made in pieces, the protein domains [10-13], with each domain showcasing its own evolutionary history. Taken together, technical and biological complexities question the validity of phylogenetic reconstructions derived from molecular sequences, especially if they are used to explore the deep evolutionary history of life. In order to overcome this limitation, it is necessary to study molecular features that are more conserved than sequences and that have evolved without major horizontal inheritance effects. Thanks to the advance of computational approaches (e.g., hidden Markov models [HMMs] and BLAST) and data integration technology, the annotation of gene products in many kinds of omics data, including genomes, transcriptomes and proteomes, has produced controlled vocabularies useful for phylogenetic analyses. These vocabularies describe molecular and functional features of organisms that are useful, such as protein structures, ontological definitions of molecular functions, the chemistries of enzymatic reactions, and connectivity of biological networks.

Several reliable classification systems of protein domains are available based on structural similarity and common evolutionary origin. For example, the Structural Classification of Proteins (SCOP) is a high-quality taxonomical resource that groups protein domains that have known three-dimensional (3D) structures into fold families (FFs), fold superfamilies and folds [10]. FFs group domains that are closely related at the sequence level (> 30% pairwise amino acid identities) or that share similar structures and functions with lower sequence identity. Fold superfamilies unify FFs that share functional and structural features, suggesting that they probably have common evolutionary origins. Finally, folds group fold superfamilies that have similar arrangements of secondary structures in 3D space but that may not be evolutionarily related due to sequence divergence. As other protein classifications, SCOP was established based on hierarchical levels of structural complexity, each of which represents a certain extent of evolutionary conservation. SCOP currently describes known structures in Protein Data Bank (PDB) entries with about 1,200 folds, 2,000 fold superfamilies, and 4,000 FFs. The relatively small numbers of these domain structures indicate that they are more conserved than domains defined by other classification schemes, such as those of the Pfam database, with levels of molecular diversity that are closer to protein sequence. A recent version of Pfam contains 11,912 distinct domains representing over 10^7 ^proteins [11]. While protein domains defined as groups of orthologous sequences share the same problems of sequence analysis, SCOP domain structures are highly conserved evolutionary units [12] that can be used effectively to uncover evolutionary patterns in the history of life [13].

As genes duplicate and diversify, ancient domain structures accumulate to larger extent in proteomes than younger structures. Although convergent evolution, horizontal gene transfer, and recruitment can occur over time, the magnitude of these processes has been shown to have little influence on the vertical inheritance of domain structures [14,15]. Their abundance in proteomes harbors deep phylogenetic signal, which can be unfolded using standard phylogenetic methods [13,16]. Global phylogenomic trees describing the evolution of domain structures can be reconstructed from a structural census [17]. This census assigns structures to genomic sequences with HMMs of structural recognition [18]. Over 10^7 ^proteins have been assigned to folds, fold superfamilies, and FFs in over 1,400 proteomes and trees of domain structures have been reconstructed at all levels of structural abstraction [17,19-21]. Work of this kind has also been extended to the evolutionary study of molecular functions and biological processes in genomes, as these are the direct consequence of protein structure [22].

The rooted trees of domain structures display in their branches the relative ancestries of domains, and these ancestries can be directly associated with chronologies of proteins, proteomes, molecular functions, biological networks, and evolutionary events of significance, such as the division of three superkingdoms and the emergence of aerobic metabolism and photosynthesis. Evolutionary studies of the protein world have been conducted primarily at the fold and fold superfamily levels [17,19,21]. However, these levels may not always guarantee common origins of domains and their associated molecular functions can be ambiguous. In this regard, revisiting the evolutionary history of the protein world at the level of FFs can be very valuable, especially because each FF is functionally orthologous and conserved enough to portray the entire history of life. Here we describe for the first time global evolutionary patterns of FFs by reconstructing phylogenomic trees of domains structures and trees of proteomes. We start with a census of 2,493 FFs in 645 proteomes of free-living organisms and facultative and obligate parasites belonging to the three superkingdoms. In our analyses we consider non-vertical evolutionary phenomena (e.g., convergent evolution, horizontal gene transfer, recruitment) as well as genome reduction. We also dissect secondary genomic reductive processes by excluding parasitic organisms. Trees describing the evolution of 2,397 FFs and 420 proteomes from free-living organisms established timelines of FFs and their associated molecular functions, which were defined using a coarse-grained functional classification [23], delimited major evolutionary phases in the protein world, and produced trees of proteomes for each of these phases showcasing varying trends in the evolution of proteins and proteomes.

## Results and Discussion

### Genomic census and trees of fold families

We have searched for controlled vocabularies that have multiple genomic occurrences and that are appropriate for surveying ancient evolutionary history. We already found that domain structures at the fold and fold superfamily levels and their domain combinations harbor phylogenetic signatures that are congruent [17,20,24-27]. Here we study the evolution of protein domains at the FF level to determine if lower levels of structural abstraction still preserve these ancient signatures. We note that our focus is on the structure of protein domains and not on how they interact with each other, within or between molecules, or with nucleic acids and other molecules of significance. The census therefore takes protein domains out of their natural molecular and cellular context.

**Figure 1:**
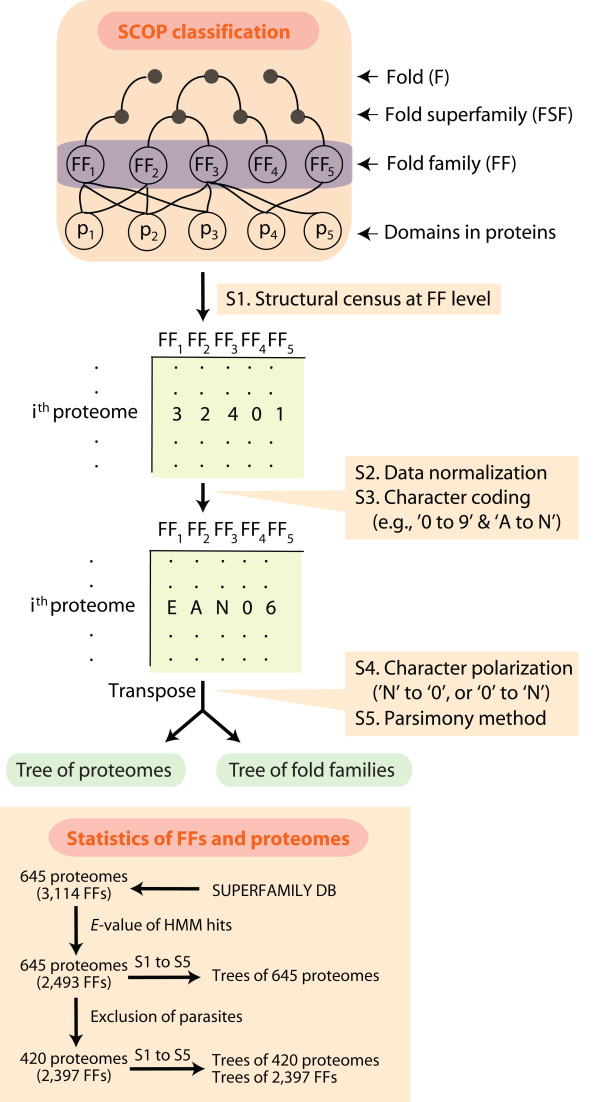
**Phylogenomic tree reconstruction at the FF level of structural classification**. The SCOP database classifies protein domains into a hierarchy of fold families (FFs), fold superfamilies and folds. In this study, we counted multiple occurrences of individual FFs in proteomes to build data matrices. Matrices of genomic abundances are normalized in a scale of '0' to '9' and 'A' to 'N' and their columns and rows can be transposed to generate both trees of proteomes and trees of FFs.

Figure 1 shows a flow diagram of our experimental strategy. The 645 completely sequenced genomes that we here analyze consist of 49 archaeal (A), 421 bacterial (B), and 175 eukaryotic (E) organisms. Manual inspection of lifestyles showed these organisms can be divided into 420 free-living (48 A, 239 B, and 133 E), 93 facultative parasitic (0 A, 71 B, and 22 E), and 132 obligate parasitic (1 A, 111 B, and 20 E) organisms. Their proteomes contained 3,114 FFs. We used an *E*-value cutoff of 10^-4 ^to extract reliable HMM hits of the FFs in individual proteomes. As a result, the structural census revealed that 2,493 FFs out of 3,114 FFs were present in the 645 proteomes. Data matrices of genomic abundance (*g*; see Methods) and genomic occurrence (presence/absence) of FFs for all possible pairs of FFs and proteomes were generated from the census. These matrices were then used to build intrinsically rooted phylogenomic trees of FF domain structures (with FFs as taxa and proteomes as characters) and trees of proteomes (with proteomes as taxa and FFs as characters) by transposing columns and rows in the matrix.

Since trees of FFs are highly unbalanced, the relative age of individual FFs can be obtained directly from the tree by counting the numbers of nodes that exist from its base to each leaf, and expressing this node index (*nd*) in a 0-1 scale (see Methods). The age of FFs derived from abundance-based trees *(nd_a_) *was strongly correlated with the age derived from occurrence-based trees (*nd*_o_) (y = 1.03 × -0.04, R^2 ^= 0.883; Additional file 1, Figure S1). While genomic occurrence of domains has been used previously to build trees of proteomes at fold superfamily level [28], a comparison of the two methods produces phyletic patterns that are largely congruent [19,24]. We thus chose to build trees of domains and trees of proteomes from FF abundance to incorporate phylogenetic signal embedded in the proteomic reuse of domains and in FFs that are widely distributed in life and had an origin that predated the last universal common ancestor (LUCA). This is not possible with an occurrence-based approach. Indeed, genomic occurrence underestimates the age of the most ancient FFs (*nd *< 0.3) (Additional file 1, Figure S1). This is expected since these FFs are widely shared and are the most abundant (see Results and Discussion below). In addition, we find that the tree of FFs based on genomic occurrence displayed a polytomy among the most ancient structural lineages (data not shown), which is fully resolved in the tree reconstructed from genomic abundance. Mechanistically, domain structures spread by recruitment as genes duplicate and diversify and genomes rearrange; their numbers are expected to increase in proteomes with evolutionary time and as species diversify. The abundance-based phylogenetic approach is therefore in line with the processes of genome evolution. Given these considerations, we here concentrate on results obtained using genomic abundance.

We note that our strategy for the construction of rooted phylogenomic trees is based on the fundamental premise that 'FFs that are more popular are more ancient'. This premise of increase representation of FFs in the protein world is not constrained by how FFs spread in the proteomes that we sample by for example gains, losses, convergent evolution, and horizontal gene transfer. In other words, our evolutionary model of tree reconstruction is not governed by the assumption that 'FFs that are more widely spread are more ancient'. While this outcome is quite frequent in our analysis, the model is agnostic about how FF growth occurs in proteomes.

### Trees of proteomes, genome reduction, and horizontal gene transfer

Reconstruction of a tree of organisms describing the evolution of 645 proteomes resulted in one most parsimonious rooted tree (Additional file 1, Figure S2). The tree was built from genomic abundances of 2,493 FFs and embodied the canonical rooting of the tree of organisms typically recovered when studying rRNA [3]. It clustered superkingdoms Archaea and Eukarya, each of which formed a monophyletic group. Bacteria was divided into two groups. One of them (group B1) was positioned at the base of the tree and contained some few bacterial facultative and obligate parasitic lineages (e.g., *Chlrorobium*, *Candidatus *Sulcia, and *Candidatus *Carsonella). In fact, the total set of 225 parasitic organisms were dispersed throughout the tree but their presence was particularly evident at the bases of their respective superkingdoms (e.g., *Giardia*, *Encephalitozoon*, etc in Eukarya; *Nanoarchaeum *in Archaea; *Mycoplasma*, *Anaplasma*, etc in group B1; see Additional file 1, Figure S2), regardless of their original taxonomic positions in rRNA trees. Parasitic organisms generally discard enzymatic and cellular machineries in exchange for resources from their hosts [19,29]. In most cases, these reductive tendencies result in small genomes and highly reduced domain repertoires. In previous studies, we found that the inclusion of these highly reduced proteomes in trees of organisms result in abnormal phylogenetic relationships [19,27]. We thus excluded proteomes from parasitic organisms and tested if their presence biased the rooting of the tree. Indeed, a tree of organisms describing the evolution of 420 proteomes of free-living organisms that was reconstructed from the abundance of 2,397 FFs (2,262 of which were parsimony-informative) showed it was rooted in Archaea (Additional file, Figure S3). Superkingdoms Bacteria and Eukarya formed monophyletic clades, each strongly supported by 100% bootstrap support (BS) values. These two superkingdoms were sister taxa to each other (53% BS) and clustered paraphyletically to archaeal proteomes, which in turn were positioned at the base of the tree. Compared with the tree of organisms that describes the evolution of the 645 proteomes, the phyletic patterns of the tree of proteomes of free-living organisms were highly congruent with those from trees of organisms built from rRNA sequences or repertoires of folds and fold superfamilies [19,24,27]. In addition, there was significant phylogenetic signal (*g_1 _*= -0.241), confirming that FF data is appropriate for deep phylogenetic studies.

While horizontal gene transfer seems rampant at sequence level, its impact appears quite limited at higher levels of structural organization [15,20,22]. We tested however if FFs evolved without major horizontal gene transfer biases. Informational genes that are involved in transcription, translation, and DNA replication have been reported to be refractory to the effects of horizontal gene transfer [30]. We therefore divided the 2,262 parsimony-informative FFs into informational (182 FFs) and non-informational (2,080 FFs) domains using as reference Vogel and Chothia's functional classification [23]. It is also well established that horizontal gene transfer occurs more frequently in Bacteria than in the other superkingdoms. We thus extracted informational (34 FFs) and non-informational (488 FFs) domains that are uniquely present in the proteomes of the 239 bacterial free-living organisms. For each of the groups, we calculated retention indexes (*r_i_*) of individual FF characters and plotted them against the age of the corresponding FFs (*nd*) derived from the tree of FF structures we describe below. The index portrays the relative amount of homoplasy of individual phylogenetic characters (conflict in how data matches the reconstructed tree) and processes other than vertical inheritance, such as convergent evolution, horizontal gene transfer and recruitment [20]. It is important to note that the measure is independent of the number of taxa in reconstructed trees. Both *r_i _*distributions for informational and non-informational FFs were highly consistent with each other and consistency was still maintained in the FFs of Bacteria (Additional file 1, Figure S4). These results do not support the argument that horizontal gene transfer is rare in informational genes since they generally interact with large number of other molecules [30]. Instead, results indicate that in contrast with sequence, horizontal gene transfer occurs with no functional preference at the FF level.

### Global evolutionary patterns of FF domain structures

**Figure 2:**
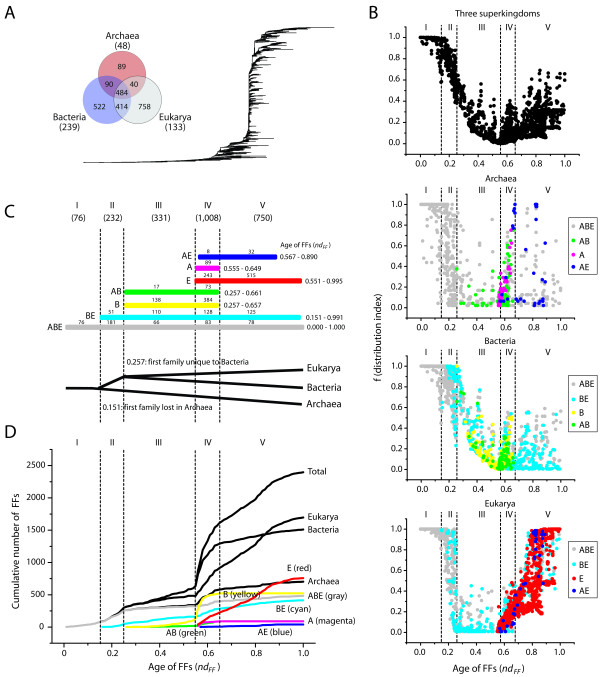
**A timeline of domain appearance in the protein world**. (A) Phylogenomic tree of FF domain structures (tree length = 177,864; CI = 0.030; RI = 0.749; *g_1 _*= -0.070) reconstructed from a genomic census of 2,397 FFs in 420 proteomes of free-living organisms (all 420 characters were parsimoniously informative). Terminal leaves are not labeled because they would not be legible. The Venn diagram shows diversity of FFs in the three superkingdoms. (B) Five phases in the evolutionary timeline of appearance of FFs in all three superkingdoms (top), and in Archaea, Bacteria, and Eukarya. Individual plots show the relationships of *f *(distribution index) and *nd *values (age of FFs). (C) The seven horizontal bars indicate *nd *ranges for taxonomic groups of FFs that are unique to individual superkingdoms (A, B, E) or shared by two (AB, BE, AE) or all (ABE) superkingdoms. The total number of FFs emerging in each phase is indicated in parentheses. The numbers labeled above bars indicate diversity of FFs belonging to taxonomic groups in each phase. (D) Cumulative frequency distribution of FFs along the timeline of domain structures.

Intrinsically rooted trees of FFs were reconstructed from the structural census of FFs in the 420 proteomes of free-living organisms we analyzed. The most parsimonious tree describing the evolution of 2,397 FFs had significant phylogenetic signal (*g_1 _*= -0.070) despite the large number of taxa (Figure 2A). We assigned relative ages of FFs (*nd*) and calculated the fraction of proteomes containing FFs (*f*; see Methods) to examine the relationship between the age and genomic distribution of domains (Figure 2B). As expected, the 13 most ancient FFs were present in all proteomes (*f *= 1), indicating that the most ancient FFs are both widely distributed and are highly conserved. However, domain loss and their distribution in emerging lineages are expected to reduce the wide distribution of domains and decrease *f *values. Indeed, the *f *values of FFs decreased with the increase of *nd *until *f *reaches 0 at about *nd *= 0.550. After this point, the pattern of change reverses and both *f *and *nd *values become positively correlated. This probably results from horizontal gene transfer, domain duplication and recruitment, and rearrangement, among other factors.

The evolutionary patterns in these plots are remarkably similar to those observed in trees of folds and fold superfamilies [19] or their domain combinations [31]. However, they are clearly apparent with lower variance of *f *values at every time point. Moreover, the global trend of *f *in the timeline can be better dissected into superkingdom-specific patterns. In the case of Archaea, the *f *values declined heavily early in time (*nd *< 0.151), reached zero at about *nd *= 0.151, rose suddenly within 0.551 ≤ *nd *≤ 0.661, an interval in which all Archaea-specific FFs (A in Figure 2B) appeared, and were dispersed in the remaining parts of the timeline. On the other hand, the trend of *f *values for Bacteria was quite similar to the global trend but showed additional features: (1) At *nd *≥ 0.151, the *f *distribution of FFs shared by Bacteria and Eukarya (BE in Figure 2B) was similar to that of FFs shared by all superkingdoms (ABE in Figure 2B); (2) The *f *values of FFs in the 0.151 ≤ *nd *≤ 0.256 interval were slightly lower; (3) FFs that were unique to Bacteria or were shared by Archaea and Bacteria (AB in Figure 2B) were only present in the 0.256 ≤ *nd *≤ 0.661 interval and showed two abnormal peaks in the distribution of *f *values at about *nd *= 0.4 and 0.6; and (4) After *nd *= 0.661, many FFs were lost (had *f *values of zero). Finally, in the case of Eukarya, the *f *values in the early part of the timeline (*nd *≤ 0.256) decreased more than those of Bacteria but less than those of Archaea. The extent of *f*-value dispersal in the 0.256 ≤ *nd *≤ 0.550 interval was highly reduced in comparison to that of Bacteria. Starting at about *nd *= 0.550, *f *values increased dramatically along the timeline. In this period, the majority of FFs are Eukarya-specific. Consequently, while loss of the domain structures occurred in all superkingdoms before the inflection point at *nd *= 0.550, a new trend in architectural innovation by gain of domains became predominant after that time.

The 2,397 FFs are not equally distributed between superkingdoms. A Venn diagram shows FFs that are uniquely present in one (taxonomic groups A, B, or E), two (BE, AB, and AE) or three (ABE) superkingdoms, with A, B and E group labels representing Archaea, Bacteria and Eukarya, respectively (Figure 2A). Only 20% of FFs are common to all superkingdoms (group ABE). Previous studies of the distribution of folds or fold superfamilies in proteomes showed the ABE group was the most abundant of all taxonomic groups [19,20]. For example, about 65% and 62% of folds and fold superfamilies belonged to this group, respectively [19]. In contrast, the number of FFs unique to Bacteria (group B) and Eukarya (group E) were larger than the group of common FFs (ABE) (Figure 2A). The clear reduction of the number of universal domain structures with lower levels of structural abstractions is expected and showcases the decreased evolutionary conservation of FFs relative to fold superfamilies and folds.

The structural timeline (0 ≤ *nd *≤ 1) can be divided into five different phases by studying the emergence, distribution and diversity of FFs (Figure 2):

(1) *A primordial (communal) protein world *(phase I; 0 ≤ *nd *≤ 0.150): In this ancient phase, domain structures diversified but were rapidly shared by the emergent cells. Proteomes of the three superkingdoms share all 76 FFs (ABE FFs). However, some FFs were lost in few proteomes (*f *< 1; Figure 2B), most notably in Archaea, indicating the start of diversification at the protein structural level. Remarkably, the ancient FFs of this phase correspond to fold superfamilies that were previously identified as being part of LUCA [27]. We believe that this phase describes the emergence of a diverse community of primordial cells that consist of genetic founders of the three superkingdoms [32]. During this phase however there were no lineages of organisms as we know of them today. Instead, selective sweeps ensured structural innovations were retained but were tolerant of considerable diversity in the emerging proteomic repertoires. Most proteins were also multifunctional. That multifunctionality is retained today in the many functions of the corresponding fold superfamilies that unify these ancient FFs [22,31].

(2) *Reductive evolution of primordial proteins *(phase II: 0.151 ≤ *nd *≤ 0.256): This phase consists of 232 FFs, many of which (181 ABE FFs) experienced reductive evolution (*f *< 1) or were completely lost (*f *= 0) in archaeal lineages (51 BE FFs that are shared by Bacteria and Eukarya) (Figures 2B and 2C). The first domains lost in Archaea were d.122.1.1 (heat shock protein 90, N-terminal domain) and d.14.1.8 (the middle domain of heat shock protein 90), which appeared at *nd *= 0.151. Consequently, this phase features the emergence of Archaea from LUCA by reductive evolution of ancient ABE FFs. The overall evolutionary trend of domain loss was higher in Archaea than in Bacteria and Eukarya. This is exemplified by significantly reduced *f *values (Figure 2B). This phase also marks the start of a slow process of diversification in superkingdom Archaea. We thus expect that many ancient though ill-defined archaeal lineages arose during this time. Since many archaeal species have adapted to extreme environments, we propose that the marked proteomic reduction of primordial archaeal species was probably caused by adaptive expansions of the LUCA into the harsh environments of early Earth.

(3) *Development of the three superkingdoms *(phase III: 0.257 ≤ *nd *≤ 0.550): Here, the ancestral lineage that is sister to Archaea gives rise to superkingdoms Bacteria and Eukarya. The primordial trend of domain loss responsible for superkingdom Archaea is still maintained (Figure 2B). FFs unique to Bacteria (138 B FFs) probably appear from loss of BE or ABE FFs. For example, the first FFs lost in Eukarya, c.40.1.1 (C-terminal domain of methylesterase) and c.116.1.4 (tRNA-methyltransferase), occurred at *nd *= 0.257 and had considerable representation in superkingdoms (*f *= 0.41 and = 0.57, respectively). This suggests that the most recent eukaryal ancestor was derived from the common ancestor of Bacteria and Eukarya. Results also exclude the possibility that Eukarya originated from Archaea, a conclusion that is also supported globally by the archaeal rooting and the sister relationship between Bacteria and Eukarya in the trees of proteomes of free-living organisms (Additional file 1, Figure S3). Consequently, the topology of the tree of proteomes should be [A, [B, E]]. Most importantly, all of the three superkingdoms reduced their proteomic complements by domain loss during this phase of superkingdom development. This is clearly evident in the substantial decrease in the appearance of FFs in the proteomes of Archaea, Bacteria and Eukarya during this phase (Figure 2D).

(4) *Organismal diversification *(phase IV: 0.551 ≤ *nd *≤ 0.661): This period embodies the 'big bang' of domain organization in proteins [31]. Despite its short time span, phase IV is responsible for over 42% of modern FFs (see the sharp slope of 'Total' in Figure 2D). At *nd *≥ 0.551, *f *values for all superkingdoms are positively (instead of negatively) correlated with *nd *values. The looser trend was therefore replaced by massive domain gains and structural innovations. A total of 1,008 FFs appear as part of all seven taxonomic groups (ABE, BE, AB, B, AE, A and E). Widespread appearance of domain structures in organismal lineages across the three superkingdoms signals massive diversification of proteins and proteomes. In addition, Archaea and Bacteria (but not Eukarya) showed abnormal peaks in the *f *distribution plots (Figure 2B) and *r_i _*values of the FFs of this phase were significantly lower than the rest (Additional file 1, Figure S4). These observations suggest that horizontal gene transfer and processes of recruitment (e.g., genome rearrangement mechanisms responsible for domain combinations) largely contributed to the make-up and diversification of the superkingdoms. For example, the appearance of 384 FFs unique to Bacteria (Figures 2C and 2D) supports the conclusion.

(5) *Eukaryal diversification *(phase V: 0.662 ≤ *nd *≤ 1): The majority of new FFs appearing in this final period were unique to the emerging eukaryotic lineages (515 out of 750 E FFs; Figure 2C). In contrast, FFs belonging to the A, AB, and B taxonomic groups were conspicuously absent, suggesting a halt of domain innovation in microbial superkingdoms. Similarly, domain appearance in the AE, BE, and ABE taxonomic groups was considerably reduced. Massive duplication of genes, genome duplications and rearrangements, meiosis, sex, and other reproductive innovations should be considered ultimately responsible for domain combination, domain recruitment and emergence of new domains in Eukarya, fundamentally by fission [31], which is typical of the most modern phase of the protein world.

### Domain diversity increases in evolution

The accumulation of FFs along the timeline shows that the numbers of different FFs always increase in the proteomes of superkingdoms despite the early and massive episodes of domain loss and the lack of appearance of new FFs specific to microbial superkingdoms in the late phases of protein evolution (Figure 2D). This observation provides support to the evolutionary model used to root the trees of proteomes, which polarizes character state changes in proteomes towards increases in genome abundance (see details in Methods).

### Evolution of molecular functions associated with FFs

**Figure 3:**
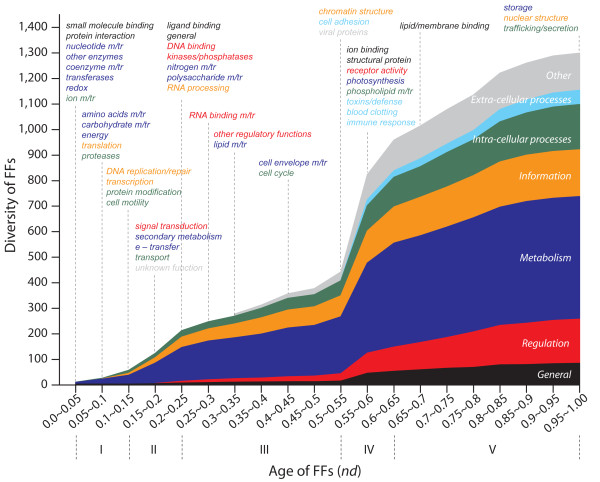
**Emergence and evolution of molecular functions along the timeline**. The cumulative frequency distribution plot illustrates the accumulation of FFs associated with the seven major functional categories of SUPERFAMILY. Dotted lines indicate the first appearance of FFs associated with one of the 49 minor functional categories.

Molecular functions are linked to corresponding protein domain structures. For the most part, structure-function relationships are unambiguous at the FF level of structural abstraction. In order to simplify the description of the functions of hundreds of FFs, we used the functional classification of Vogel and Chothia [23]. A total of 1,299 FFs were grouped into one of 7 major categories (*General*, *Information*, *Metabolism*, *Intra-cellular processes*, *Extra-cellular processes*, *Regulation*, and *Other*) and into one of 49 minor categories of molecular functions. For simplicity, the names of the categories were displayed in italics and the initial letters of the major categories were capitalized. The emergence time points of the major and minor categories of molecular functions in the timeline of FFs revealed remarkable patterns of origin in the five evolutionary phases (Figure 3).

Phase I: Only three of the seven major categories were present very early in the FFs of phase I. They included minor categories *small molecule binding *and *protein interaction *of *General*, *ion m/tr (m*/tr stands for metabolism and transport) of *Intra-cellular processes*, and *nucleotide m/tr*, *other enzymes*, *coenzyme m/tr*, *transferases*, and *redox *of *Metabolism*. Since *small molecule binding *and *ion m/tr *involve popular multifunctional enzymes and membrane transporters (e.g., ATPases), the vast majorities of molecules emerging at the beginning of modern cellular life were involved in making up modern metabolic enzymes and enabling transport processes across primordial membranes. This suggests primitive cells acted as containers of the emerging protein domains already during this first evolutionary phase. The notable absence of molecular functions involved in *Information *indicates that ancient catalytic proteins with primordial metabolic functions initiated life in the absence of a translational apparatus. This conclusion is supported by the mapping of functions in a timeline of fold superfamilies [13,19] and by phylogenomic analyses of structures and functional ontologies [20,22]. The minor categories *translation *(*Information*), *amino acids m/tr*, *carbohydrate m/tr*, and *energy *(*Metabolism*), and *proteases *(*Intra-cellular processes*) appeared for the first time very late in phase I. The first FFs of translation were the catalytic domains of aminoacyl-tRNA synthetases [20]. Thus, translation emerges after crucial metabolic activities and together with amino acids biosynthesis and polypeptide breakdown [20,22]. Results once again support the metabolism-first hypothesis of the origin of life and refute the existence of an RNA world (see [20] for an extended discussion and [33] for a review).

Phase II: This period starts with the emergence of FFs belonging to *DNA replication/repair *and *transcription *(*Information*), suggesting that early during this time nucleic acids started to be used as genetic repository. In addition, the appearance of *protein modification *and *cell motility *(*Intra-cellular processes*) suggests the start of cellular diversification. Late in phase II, functions related to *signal transduction *(*Regulation*), *secondary metabolism *and *e-transfer *(electron transfer) (*Metabolism*), and *transport *(*Intra-cellular processes*) suggest more advanced cellular systems capable of regulatory control of cellular processes and more efficient energy management.

Phase III: With the exception of *Extra-cellular processes *and *Other*, all major categories are represented in this period and include *ligand binding *and *general *(*General*), *DNA binding*, *kinases/phosphatases*, *RNA binding m/tr *and *other regulatory functions *(*Regulation*), *nitrogen m/tr*, *polysaccharide m/tr*, *lipid m/tr *and *cell envelope m/tr *(*Metabolism*), *RNA processing *(*Information*) and *cell cycle *(*Intra-cellular processes*) (Figure 3). Functions such as *lipid m/tr *and *cell envelope m/tr *emerged quite late in the period and are clearly associated with the rise of superkingdoms Bacteria and Eukarya (the fundamental feature that defines this phase) (Figure 2C). For example, FFs involved in these processes established the chirality and chemistry of glycerol membranes by diversifying primordial ether and ester lipids that were present in LUCA into the *sn2,3 *isoprenoid ether lipids of Archaea and the *sn1,2 *fatty acid ester lipids of Bacteria and Eukarya [27]. Remarkably, molecular functions and FFs withered as the phase progressed and in preparation of a truly diversified world of organisms approaches.

Phase IV: The molecular functions added in this relatively short phase of protein and proteomic diversification start with *chromatin structure *(*Information*), *cell adhesion *(*Extra-cellular processes*), and *viral proteins *(*Other*), and are followed by *ion binding *and *structural protein *(*General*), *receptor activity *(*Regulation*), *photosynthesis *(*Metabolism*), *phospholipid m/tr *(*Intra-cellular processes*), and *toxins/defense*, *blood clotting *and *immune response *(*Extra-cellular processes*). These functions are quite advanced and involve complex variants of Bacteria and Eukarya that engage in multicellularity, cell communication, and interaction with the environment at various biological levels (e.g., between cells or among organisms).

Phase V: This final phase has the longest time span but introduced only four functional innovations: *lipid/membrane binding *(*General*), *storage *(*Metabolism*), *nuclear structure *(*Information*), and intracellular *trafficking/secretion *(*Intra-cellular processes*). All of these processes are involved in establishing a much more complex cellular structure, such as the formation of compartments (e.g., the nucleus), lipid and polysaccharide storage, and targeting of proteins to proper compartments, sorting and translocation, and protein secretion mechanisms. All of these innovations are quite elaborated in Eukarya and involve many of Eukarya-specific FFs that appear abundantly in this phase.

### Phase-specific trees of proteomes along the timeline

**Figure 4:**
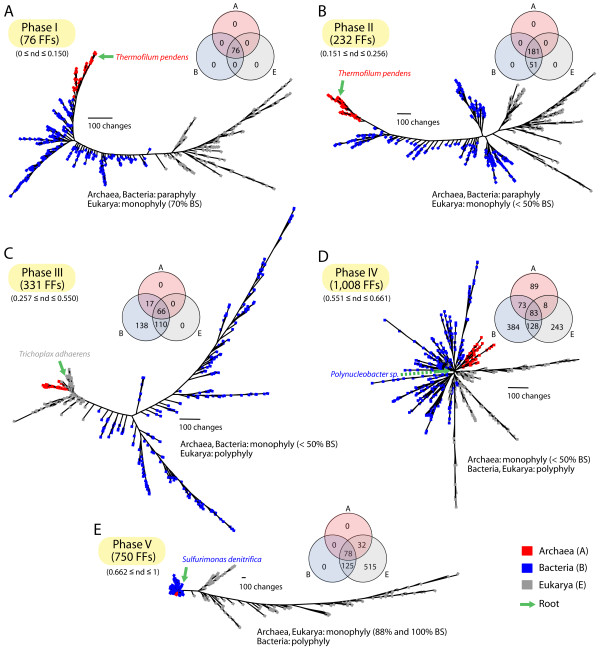
**Species trees reconstructed using phase-specific FFs in 420 proteomes of free-living organisms**. (A) Tree of proteomes reconstructed from the 76 FFs of phase I (tree length = 12,829 steps; CI = 0.065, RI = 0.727; *g_1 _*= -0.116). (B) Tree of proteomes reconstructed from the 232 FFs of phase II (tree length = 28,447 steps; CI = 0.056, RI = 0.708; *g_1 _*= -0.242). (C) Tree of proteomes reconstructed from the 331 FFs of phase III (tree length = 13,773 steps; CI = 0.082, RI = 0.783; *g_1 _*= -0.212). (D) Tree of proteomes reconstructed from the 1,008 FFs of phase IV (tree length = 21,804 steps; CI = 0.153, RI = 0.614; *g_1 _*= -0.356). (E) Tree of proteomes reconstructed from the 750 FFs of phase V (tree length = 47,213 steps; CI = 0.136, RI = 0.828; *g_1 _*= -0.284). Terminal leaves of Archaea, Bacteria, and Eukarya were labeled in red, blue, and gray. Venn diagrams show the distribution of FFs in the three superkingdoms for the FFs of each phase. The green arrow indicates the root position of a given tree of proteomes.

In order to examine how the deep phyletic patterns of the three superkingdoms changed along the timeline, we reconstructed trees of proteomes for each of the five evolutionary phases (Figure 4). Again, we avoided the relatively modern reductive effects of parasitism by extracting phase-specific FFs present in the set of proteomes of free-living organisms. The main assumption in these studies is that different phases carry FFs with different phylogenetic signatures that describe selected aspect of life's evolution.

The most parsimonious tree of proteomes for phase I was reconstructed using genomic abundances of the universal 76 ABE FFs that appeared during the 0 ≤ *nd *≤ 0.150 time interval (Figure 4A). The tree shows that the three superkingdoms formed separate groups. Proteomes of Archaea and Bacteria appeared paraphyletic while proteomes of Eukarya formed a moderately supported (70% BS) monophyletic group. The tree was rooted in Archaea, which was positioned at its base. *Thermofilum pendens*, a hyperthermophilic archaeon belonging to the phylum Crenarchaeota, was the most basal taxon. On the other hand, bacterial proteomes spanned the ancient archaeal lineages and the more derived eukaryal counterparts. The timeline derived from the tree of FFs shows no separation of the three superkingdoms in this phase, since all FFs of this phase are common to all life (Figure 2C). However, the phylogenetic signal embedded in the genomic abundances of these very old FFs, which contain domains of all ages in their make-up (the 'modern effect' sensu [27]), is strong and dissects the appearance of the three superkingdoms. The archaeal root of the tree of proteomes that is apparent already in phase I is consistent with the first emergence of Archaea from LUCA in the timeline of domain structures (Figure 2C). Remarkably, the tree of proteomes reconstructed from genomic abundances of the 181 ABE and 51 BE FFs of phase II is congruent with the tree reconstructed from phase I-specific FFs (Figure 4B). The tree is rooted in Archaea and shows Eukarya as a weakly supported (< 50% BS) monophyletic group. Interestingly, the most ancient 19 archaeal lineages of the phase I and phase II tree, including the *T. pendens *root, are thermophiles and hyperthermophiles and are consistently followed by methanogenic archaeal lineages in both trees. These basal topologies that are congruently recovered from trees reconstructed from the most ancient protein domain characters lend support to the hypothesis of a thermophilic bottleneck during the rise of diversified lineages.

However, the deep relationships of the three superkingdoms present in phases I and II are broken in the tree of proteomes reconstructed from genomic abundances of the 331 FFs (66 ABE + 110 BE + 17 AB + 138 B FFs) of phase III (Figure 4C). Bacterial proteomes now clustered monophyletically and eukaryotic species formed a polyphyletic group at the base of the tree that included a monophyletic archaeal group. The eukaryotic placozoan *Trichoplax adhaerens *roots the tree of proteomes. It is also noteworthy that distributions of branch lengths show high levels of divergence in Bacteria in this phase when compared to the basal Archaea and Eukarya. The many bacteria-specific FFs of this period provide further support to the existence of high levels of bacterial diversification. The tree of proteomes reconstructed using the 1,008 FFs of phase IV that belong to all seven taxonomic groups was star-like and was rooted in a β-proteobacterium *Polynucleobacter *sp. (Figure 4D). Most lineages in the three superkingdoms formed polytomies. Bacterial and eukaryal species were polyphyletic. Instead, archaeal species formed a poorly supported clade. The star-like tree suggests horizontal gene transfer occurred rampantly across the three superkingdoms (also supported by peaks of *f *distribution in Figure 2B). Finally, the tree of proteomes reconstructed from the 750 eukaryotic FFs (78 ABE, 32 AE, 125 BE, and 515 E FFs) of phase V supported monophyletic Archaea and Eukarya and was rooted in Bacteria. However, the archaeal group bisected bacterial groups. Unlike the trees of proteomes for the other previous four phases, eukaryal lineages were highly divergent, indicating that duplication of genes and genomes has frequently occurred in eukaryal lineages.

The canonical rooting of the tree of organisms derived from phylogenetic analyses of rRNA and other sequences (e.g., ATPases, aminoacyl-tRNA synthetases, elongation factors) generally shows hyperthermophilic bacteria (e.g., Thermotogae) at the base of the tree [32]. Our results do not support this topology. Instead, results are compatible with the hypothesis that the tree of organisms is rooted in an ancestor of modern archaeal proteomes [22,27]. The archaeal rooting has been reliably obtained in numerous studies with different proteomic sets [13,22,24,27] and is congruent with results from phylogenetic analysis of the structure of tRNA [34,35], 5S rRNA [36] and RNase P [37], and of tRNA paralogs [38-41]. Remarkably, a molecular clock of folds also revealed that the first fold lost in a superkingdom disappeared in Archaea 2.6 billion years ago, within the span of the rise of planetary oxygen that preceded the great oxidation event on Earth [21]. Similarly, a careful reconstruction of the fold superfamily repertoire of LUCA showed it emerged sometime between 2.9 and 2 billion years ago, after the development of primordial ribosomal protein synthesis [27]. Trees of proteomes reconstructed from FFs appearing in the five evolutionary phases of domain diversification and from the entire set of FFs now confirm the archaeal rooting of diversified life.

### Growth of FF repertoires in proteomes

**Figure 5:**
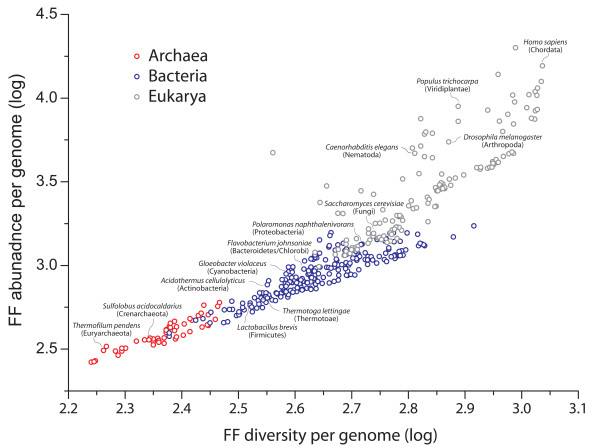
**Plots of use and reuse of FFs in proteomes**. The sum of multiple occurrences of FFs was plotted against the number of distinct FFs for each of 420 proteomes of free-living organisms, including 48 Archaea (red circles), 239 Bacteria (blue circles), and 133 Eukarya (gray circles). Both axes are in logarithmic scale.

Plots describing the evolutionary accumulation of FFs in proteomes that were directly derived from the intrinsically rooted trees of FFs (Figure 2D) show that domain gains always overwhelm domain loss. Moreover, they show that the repertoires of FFs always increase in all superkingdoms and in all taxonomic groups of FFs, regardless of the strong reductive evolutionary trends identified in the timelines. Even FF repertoires of individual free-living organisms exhibit these same trends. This overwhelming tendency of domain growth in proteome evolution that occurs throughout the timeline (regardless of how widely shared are FFs in the protein world) supports the character polarization statements that we use to root the trees of proteomes, and falsifies any character polarization scheme that may be applied in an opposite direction for the reconstruction of trees of proteomes. This trend, in conjunction with strong reductive evolutionary episodes of domain loss that occur in Archaea, Bacteria and Eukarya, also dissects the three superkingdoms in representations of occurrence and abundance of FFs in proteomes. A simple 'non-historical' plot of use of FFs (number of distinct FFs in a proteome; i.e. FF diversity) versus reuse of FFs (the sum of multiple occurrences of FFs in a proteome; i.e., FF abundance) shows a clear increase in values for the individual proteomes analyzed, starting with the proteomes of Archaea, then those of Bacteria, and finally those of Eukarya (Figure 5). Besides dissecting the three superkingdoms without phylogenetic reconstruction and supporting our character polarization statements, these patterns, in conjunction with the results of Figure 2, suggest the plot of FF use and reuse (Figure 5) should be interpreted as a temporal progression of proteome appearance. The index of proteomes in the figure indeed confirms that both axes of the plot are correlated with evolutionary time and reveals once again the temporal progression of Archaea, followed by Bacteria and Eukarya.

Protein structures are unevenly distributed in the world of proteins and proteomes [13]. Genomic surveys reveal they follow power-law distributions and establish networks with scale-free properties. This shows a preference for duplication of genes encoding protein structures that are already common--a "rich get richer" process, which we here use to root our trees of FFs. Interestingly, frequency plots of fold structures for microbial superkingdoms Archaea and Bacteria had steeper slopes that those of Eukarya, showing folds accumulate at higher rates in the proteomes of complex organisms [17]. However, the most ancient folds that are shared by all organisms or are shared by Bacteria and Eukarya fitted Gaussian-like distributions characteristic of random graphs, suggesting the spread of these structures across superkingdoms is complex [17]. Figure 5 uncovers the interplay between forces that produce redundancy (e.g., gene duplication) and forces that degrade it (e.g., mutation), an interplay that is ultimately responsible for the rise and diversification of FF structural modules. In contrast to redundancy, modularity can spread pervasively in genomes, increasing their size and slowing down replication time and proliferation. Consequently, the costs of limited proliferation curb excessive increases in modularity, especially in r-selected organisms such as those of microbial superkingdoms, which can only pack a limited gene repertoire in their genomes and thrive in competitive environments. In contrast, K-selected organisms such as eukaryotes can tolerate module expansion within confines of rates of error correction in DNA replication and growth conditions dictated by the environment.

## Conclusions

**Figure 6:**
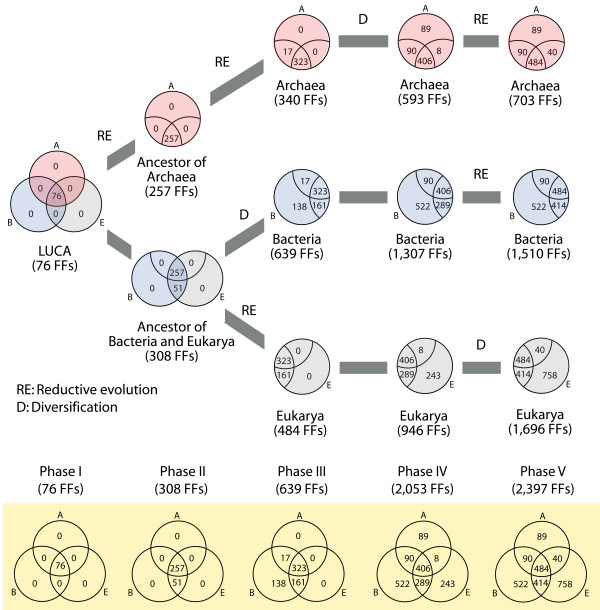
**Model of protein domain and proteome diversification**. Venn diagrams show the evolutionary accumulation of FFs in superkingdoms as these distribute in the five phases of the timeline. The tree diagram in the top describes major dichotomies in the organismal world and shows how reductive evolution and diversification has tailored proteome evolution in the three cellular superkingdoms. The model was assembled directly from phylogenomic data.

Protein functions are tightly coupled with protein structures and structures are much more conserved than sequences [13]. This makes structures 'molecular fossils', 'canalized' remnants of ancient organization in biological molecules. In this study we show that protein domains studied at the FF level of abstraction hold deep phylogenetic information and at the same time are varied enough to be linked for the most part unambiguously to the functions of molecules. We also show FFs dissect the history of proteins and proteomes in five evolutionary phases, each of which portrays clear episodes of molecular and cellular diversification. Reductive evolutionary processes of domain loss dominate the first three phases while the last two favor the gain rather than the loss of domains. While these trends were already visible in the analyses of folds and fold superfamilies [19], phylogenomic analysis of FFs now reveals clear historical patterns of diversification of organisms, structures and molecular functions. The taxonomic distributions of domains in superkingdoms as they appear in the different phases uncover the history of the tripartite world of organisms (Figure 2). Remarkably, these evolutionary patterns are confirmed by the reconstruction of species trees from the proteomes of the free-living organisms analyzed, despite the limitations that trees of organisms impose on phylogenetic reconstructions (Figures 2 and 4). Accumulation of FFs in superkingdoms as the timeline of FF appearance unfolds and Venn diagrams for each phase describing the accumulation of FFs in the protein world show striking patterns (Figure 6): (i) the structural diversification of LUCA in phase I to produce a functionally complex cellular ancestor of life (see also [27]), (ii) the rise of Archaea in phase II by loss of FFs and appearance of BE FFs, signaling the first dichotomy from LUCA that generates the first lineages of Archaea and the ancestor of Bacteria and Eukarya, (iii) the rise of Bacteria from the common ancestor of Bacteria and Eukarya in phase III, exemplified by the appearance of Bacteria-specific B FFs, signaling the second dichotomy from the common ancestor of Bacteria and Eukarya that generates Bacteria and the ancestor of Eukarya, (iv) the rise of modern Eukarya from the common ancestor of eukaryotic lineages and of modern Archaea from ancient archaeal lineages in phase IV, exemplified by the appearance of Eukarya and Archaea-specific E and A FFs, and (vi) the diversification of Eukarya exemplified by the massive and exclusive appearance of eukaryotic FFs in phase V. These results place the ahistorical analysis of taxonomic distributions of fold superfamilies [17], previously explained by the parsimony rationale [42], into a phylogenomic-based historical context. The structural genomic census can now be framed with a model of evolution that dissects unequivocally the first appearance of lineages and their diversification in the tree of organisms. We must conclude from this genomic-driven model that the microbial superkingdoms arose gradually by evolutionary loss of FFs as they diversified in proteomes, that ancient archaeal organisms were the first cellular lineages derived from LUCA, that these lineages diversified slowly and manifested fully as Archaea very late in evolution, that Bacteria appeared later but diversified relatively quickly, and that Eukarya was the last to fully materialize as a superkingdom. Our model now reconciles the canonical view of a bacterial-like origin of life with a functionally complex eukaryotic-like LUCA and the ancient and gradual rise of the ancestors of Archaea suggested by paleobiological and phylogenomic evidence [21,27]. Remarkably, our model of early archaeal emergence < 2.9 billion years ago derived from a molecular clock of folds [21,27] is congruent with the recent proposal that diversification of lineages in Archaea was very gradual and began 2.8 billion years ago while impacting fundamental biogeochemical S, C, and N cycles [43].

## Methods

### Assigning FFs to proteomes

We downloaded the local MYSQL database from SUPERFAMILY ver. 1.73 [44] that assigned all known FFs to proteomes. At the time of this analysis, the genomes of the 645 organisms we analyzed were completely sequenced. SUPERFAMILY has built HMMs for all fold superfamilies that have been defined in SCOP. Proteomes deposited in the database were scanned with the HMMs using the iterative Sequence Alignment and Modeling System (SAM) method [45], which has generated fold superfamily assignments covering ~60% of amino acid residues of individual proteomes on the average [44]. Subsequently, protein domains in individual fold superfamilies are assigned to corresponding FFs using a hybrid method that compares the two profile alignments: (1) protein domains to fold superfamily HMMs; and (2) ASTRAL reference sequence of FF to fold superfamily HMMs [46]. FF assignments that meet the *E*-value of 10^-4 ^were extracted from the individual proteomes. This *E*-value cutoff is optimal to maximize the rate of true positives in the HMM searches [46]. FFs were named using SCOP *concise classification strings *(*ccs*) (e.g., c.67.1.4, where c indicates the protein class, 67 the fold, 1 the fold superfamily, and 4 the FF). The lifestyles of the 645 organisms were manually determined based on various resources including public databases and literature review. Organisms were classified into free-living, facultative parasite, and obligate parasite categories.

### Phylogenomic analysis

According to SCOP, protein sequences that have sequence identity of over 30% or that share a common ancestor in terms of structures and functions are grouped into FFs [10,18]. Individual FFs are expected to be present multiple times in a proteome. We thus counted how many times individual FFs were assigned to each of the sampled proteomes. Here, the number of multiple domain occurrences was defined as a genomic abundance (*g*) value. A two-dimensional data matrix was constructed by calculating *g *values for all pair-wise combinations of proteomes and FFs. Empirically, *g *values ranged from 0 to hundreds and were normalized using the following formula [24],

$${\text{g}}_{ab\text{\_}norm}=Round\left[\frac{\text{ln}\left({\text{g}}_{ab}+1\right)}{\text{ln}\left({\text{g}}_{max}+1\right)}\times 23\right]$$

, where *g_ab _*describes the *g *value of FF *a *in proteome *b *and *g*_max _indicates the maximum *g *value in the matrix. The round function normalizes a *g *value for a particular FF in a proteome relative to the *g_max_*, and standardizes values to a 0-23 scale. The 24 transformed values in the matrix were linearly ordered to discrete character states using an alphanumeric format of numbers (0-9) and letters (A-N) that are compatible with the phylogenetic package PAUP* ver. 4.0b10 [47].

Phylogenomic trees of domain structures at FF level of structural abstraction were reconstructed from the data matrix of genomic abundances (multiple occurrences of FFs) using maximum parsimony (MP) with 1,000 replicates of random taxon addition, tree bisection reconnection branch swapping, and maxtrees unrestricted. In addition, we generated trees of FFs from presence/absence of FFs (FF content) to compare phyletic patterns between the two approaches: abundance and content. The character states in the matrix were polarized from 'N' to '0' using the ANCSTATES command of PAUP*, where 'N' and '0' indicate the most ancient and recent character states, respectively. High genomic abundance is considered the ancestral character state because domains that are ancient had more time to accumulate in proteomes and to spread in the world of proteins than domains that have a more recent origin. Note that this is not a proteome-specific statement but a global statement, especially because trees of FFs describe the evolution of the protein world. Moreover, there is no cap in the growth of domains (imposed for example by the energetic costs of their replication) since they can be unpopular in one lineage but popular in another. In other words, their numbers can increase without constraints as long as they can be accommodated with major costs in a lineage or can be apportioned in different lineages. In summary, polarization refers to character state change, a property of characters that affect evolution of taxa. Characters are proteomes, which technically are infinite in number and for the most part evolve independently from each other (if lineages are taxonomically distant). Taxa are FFs, proteomic parts that are finite in number and can grow unabated in proteomes. We thus claim no constraints in our model of FF evolution.

Phylogenomic trees of proteomes were reconstructed after transposing the data matrix and polarizing character states from '0' to 'N', with '0' being ancestral. Low genomic abundance is thus considered the ancestral character state because we expect that proteome size will increase by the repeated accumulation of domains (via gene duplication and mutational diversification). Under this model, the primordial proteome contained a handful of domain structures that were rarely reused. With time, increases in domain diversity and reuse cause protein repertoires to enlarge, with the addition of each additional FF taking precious space in the limited proteomic make-up. Here, the energetic constraints of maintaining and replicating domains in proteomes limit proteome expansion. Consequently, there is a cap as proteomes have a finite space to accommodate domains (and their variants) and this space is "canalized" in evolution. In summary, polarization refers to characters that are parts of proteomes (FFs) that for the most part do not evolve independently from each other (given biological networks). Taxa are proteomes, technically infinite in number, but constrained by the numbers of FFs they can hold. Consequently, proteomes cannot grow unabated, especially when the number of parts increases and parts appear gradually in evolution. This limitation sets the pace of proteome growth, which in the cumulative plots of Figure 2D shows domain gain always overwhelms domain loss, regardless of the superkingdom or taxonomical group of FFs that is considered. Similarly, plots of use and reuse of fold superfamilies show a clear increase in values for proteomes, starting with Archaea, then Bacteria, and finally Eukarya [48]. We reveal these same patterns if the study of FFs. These observations support our character polarization model.

We note that when polarizing trees of FFs or trees of proteomes, our model of evolution allows for both increases and decreases in genomic abundance, enabling processes of reductive evolution and of expansion to unfold in the phylogenies. Our model does not force tree reconstructions to fit patterns of distribution in the organismal world (such as distributions of FFs in superkingdoms or organismal groups). Instead, these arise naturally from the phylogenetic reconstructions. The rationale for character coding and polarization as well as the discussion of the robustness of phylogenetic assumptions can be found elsewhere [17,25-27].

Phylogenetic confidence was evaluated by BS values [49] and the extent of phylogenetic signal was measured using the tree skewness (*g_1_*) test [50]. The consensus of the most parsimonious trees was obtained using the Python library SumTrees with the option of 50% majority rule [51]. Our phylogenetic strategy uses the Lundberg method [52] to generate rooted phylogenomic trees without the need of outgroups. The method roots the tree with a hypothetical ancestor whose attachment to an internode of the tree makes the tree most parsimonious. Therefore, the internode that is connected by the hypothetical ancestor needs to be a branch in which a plesiomorphic character appears. Consequently, the phylogenetic position of the hypothetical ancestor depends on character polarization and the character state transformation series. Empirically, the trees are rooted by the internode of terminal nodes whose characters have fewest steps to reach the ancestral character state regarding the direction of character transformation. Trees were visualized using Dendroscope ver. 2.7.4 [53]. When reconstructing trees of proteomes, retention indexes (*r_i_*) were calculated for individual FF characters with the "DIAG" (character diagnostics) command of PAUP*.

### Relative age of FFs and their distribution in proteomes

Since trees of domain structures are intrinsically rooted and highly unbalanced, we calculated the relative evolutionary age of FF taxa by counting internal nodes in the tree between the hypothetical root and a terminal node on a 0-1 scale. The node distance (*nd*) was calculated using the following formula: *nd_α _*= (# of internal nodes between nodes *r *and *α*)/(# of internal nodes between nodes *r *and *β*), where *α *represents a target terminal node, *r *is a hypothetical root node, and *β *is a terminal node that has the largest number of internal nodes from the root node. Consequently, the *nd *value of the most ancestral taxon is 0, whereas that of the most recent one is 1. Node distance can be a good measure of age given a rooted tree of FFs because the semipunctuated emergence of protein domains (i.e., taxa) is displayed by their ability to diverge (cladogenesis or molecular speciation) rather than by the amount of character state change that exists in branches of the tree (branch lengths) and is supported by the existence of a molecular clock of protein structures [21]. In addition, we calculated an index (*f*) that describes the fraction of proteomes that harbor a certain FF in a 0-1 scale. An *f *value of 0 implies the absence of that FF and a value of 1 its presence in all proteomes considered.

### Functional annotations of FFs

According to SCOP, each FF has a single molecular function. Since we deal with over 2,000 FFs, displaying the large number of functions individually is not an effective way to describe global evolutionary patterns of molecular functions. We thus used the coarse-grained classification of molecular functions of SUPERFAMILY [23], which confers tens of functional groups linked to known fold superfamilies. Although the classification is only centered on the functions of fold superfamilies, it is natural that the functional category of a particular FF should be the same to that of its parent fold superfamily. Based on this premise, we identified fold superfamilies linked to FFs with SCOP and subsequently determined the functional categories of FFs. A detail description of major and minor molecular functions can be found at http://supfam.cs.bris.ac.uk/SUPERFAMILY/function/scop.larger.categories.

## Abbreviations

BS: bootstrap support; FF: fold family; HMM: hidden Markov model; LUCA: last universal common ancestor; *nd*: node distance; SCOP: structural classification of proteins.

## Competing interests

The authors declare that they have no competing interests.

## Authors' contributions

Both authors designed experiments, analyzed data, and wrote, read and approved the manuscript.

## Supplementary Material

Additional file 1 **Figure S1. Comparison of timelines of appearance of domain structures derived from trees of FFs that were generated from either the abundance or occurrence of FFs in proteomes**. A linear correlation is significant and was displayed with a red line. **Figure S2. A phylogenomic species tree generated from the entire dataset of proteomes. The phylogram of the most parsimonious rooted tree **(tree length = 15,597 steps; CI (consistency index) = 0.083; RI (retention index) = 0.771; *g*_1 _(tree skewness) = -0.288) **describes the evolution of 645 proteomes and was generated from genomic abundances of 2,493 FFs **(2,352 of which represent parsimony informative sites). Terminal nodes of Archaea (A: 49 proteomes), Bacteria (B: 421), and Eukarya (E: 175) were labeled in red, blue, and gray, respectively. The dotted lines explicitly display the borders between two superkingdoms. The life-styles of proteomes were displayed using a vertical bar beside their terminal leaves. Proteomes from free-living (420 proteomes), facultative parasitic (94), and obligate parasitic (131) organisms were labeled in blue, red, and cyan, respectively. **Figure S3. A phylogenomic tree of proteomes describing the evolution of free-living organisms**. A most parsimonious rooted tree (tree length = 128,371 steps; CI = 0.103; RI = 0.760; *g*_1 _= -0.241) was reconstructed from the genomic abundances of 2,397 FFs in 420 proteomes (2,262 of which represent parsimony informative sites). Terminal nodes of Archaea (A: 48 proteomes), Bacteria (B: 239 proteomes), and Eukarya (E: 133 proteomes) were labeled in red, blue, and gray, respectively. BS values > 50% are shown above or below branches that cluster superkingdoms much higher groups. A Venn diagram shows the diversity of FFs in the three superkingdoms. **Figure S4. The extent of homoplasy exhibited by phylogenomic characters (FFs) in trees of proteomes**. The retention index (*r_i_*) of the FFs was determined from the tree of FL proteomes described in Figure 4. *r_i _*values for the 2,262 parsimony-informative FFs (A) and 522 bacteria-specific FFs (B) were plotted against *nd *values obtained from a tree of FFs that is described in Figure 2A. A total of 182 and 34 out of the 2,262 and 522 FFs, respectively, were involved in informational cellular processes such as transcription, translation, and DNA replication (closed symbols). Click here for file
