# Supplementary material for: The evolutionary history of protein fold families and proteomes confirms that the archaeal ancestor is more ancient than the ancestors of other superkingdoms
Source: BMC Evol Biol. 2012 Jan 27;12:13. doi: 10.1186/1471-2148-12-13 (PMC3306197; doi:10.1186/1471-2148-12-13)
Supplement: Additional file 1 — Figure S1. Comparison of timelines of appearance of domain structures derived from trees of FFs that were generated from either the abundance or occurrence of FFs in proteomes. A linear correlation is significant and was displayed with a red line. Figure S2. A phylogenomic species tree generated from the entire dataset of proteomes. The phylogram of the most parsimonious rooted tree (tree length = 15,597 steps; CI (consistency index) = 0.083; RI (retention index) = 0.771; g1 (tree skewness) = -0.288) describes the evolution of 645 proteomes and was generated from genomic abundances of 2,493 FFs (2,352 of which represent parsimony informative sites). Terminal nodes of Archaea (A: 49 proteomes), Bacteria (B: 421), and Eukarya (E: 175) were labeled in red, blue, and gray, respectively. The dotted lines explicitly display the borders between two superkingdoms. The life-styles of proteomes were displayed using a vertical bar beside their terminal leaves. Proteomes from free-living (420 proteomes), facultative parasitic (94), and obligate parasitic (131) organisms were labeled in blue, red, and cyan, respectively. Figure S3. A phylogenomic tree of proteomes describing the evolution of free-living organisms. A most parsimonious rooted tree (tree length = 128,371 steps; CI = 0.103; RI = 0.760; g1 = -0.241) was reconstructed from the genomic abundances of 2,397 FFs in 420 proteomes (2,262 of which represent parsimony informative sites). Terminal nodes of Archaea (A: 48 proteomes), Bacteria (B: 239 proteomes), and Eukarya (E: 133 proteomes) were labeled in red, blue, and gray, respectively. BS values > 50% are shown above or below branches that cluster superkingdoms much higher groups. A Venn diagram shows the diversity of FFs in the three superkingdoms. Figure S4. The extent of homoplasy exhibited by phylogenomic characters (FFs) in trees of proteomes. The retention index (ri) of the FFs was determined from the tree of FL proteomes described in Figure 4. ri values for the 2, [file 1471-2148-12-13-S1.PDF]

Additional file

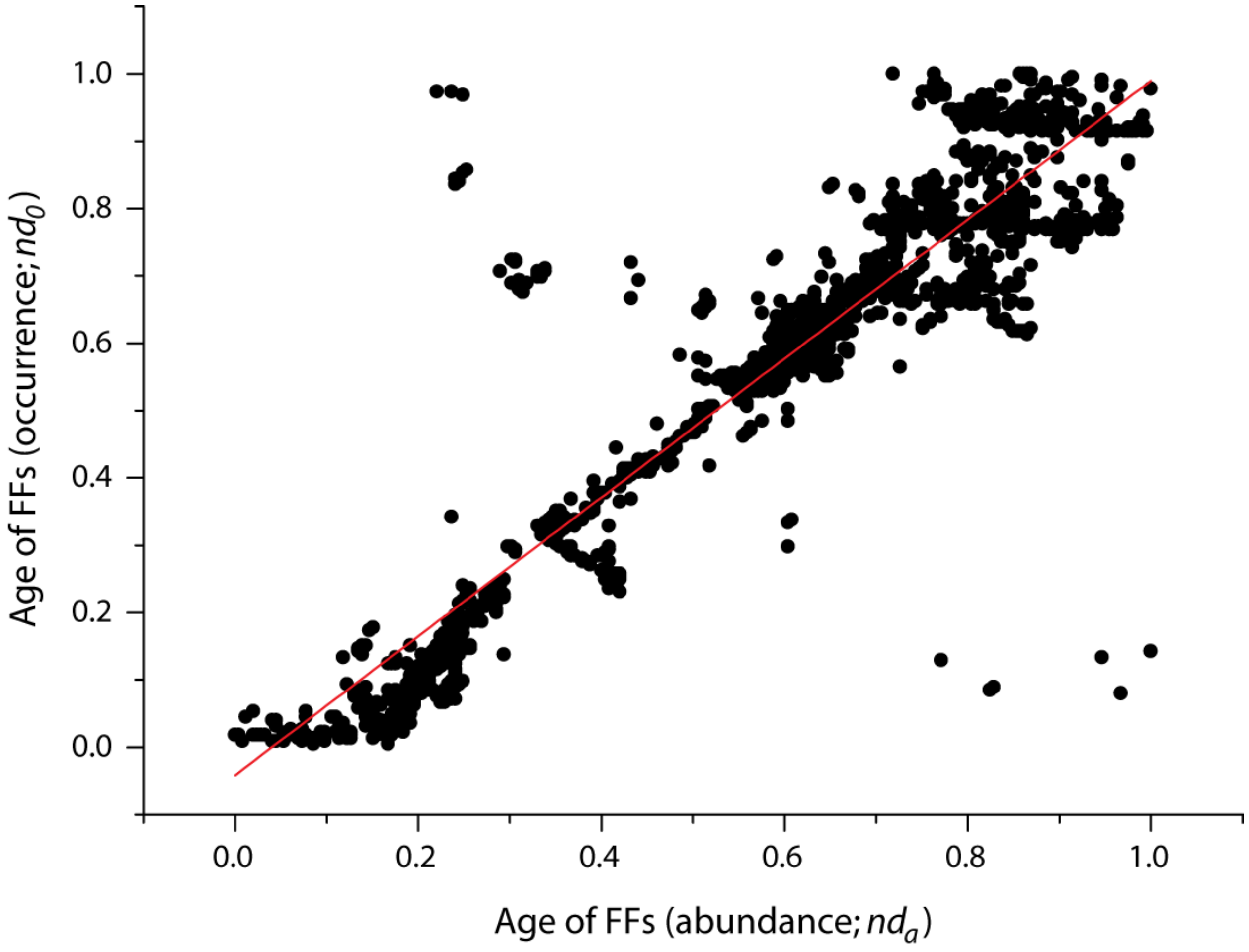

Figure S1. **Comparison of timelines of appearance of domain structures derived from trees of FFs that were generated from either the abundance or occurrence of FFs in proteomes.** A linear correlation is significant and was displayed with a red line.

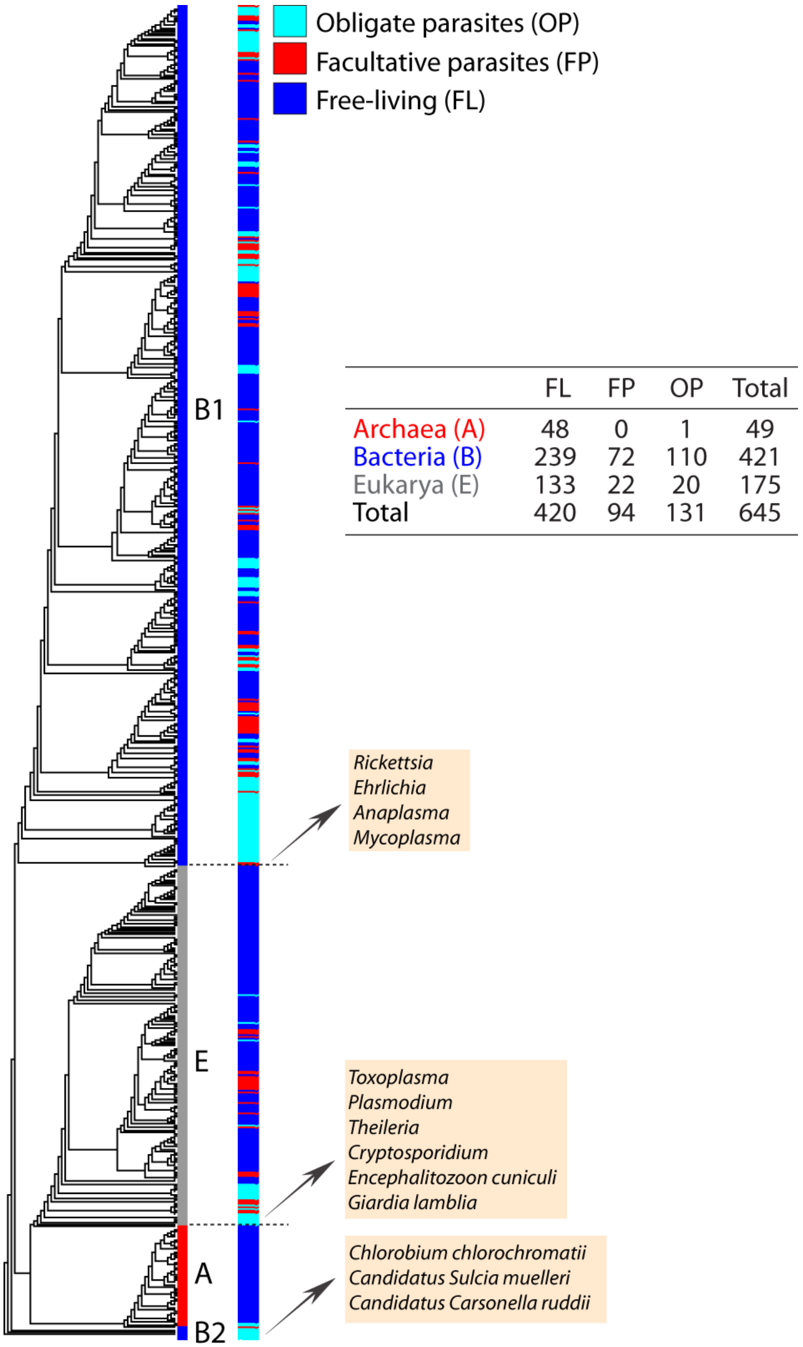

	FL	FP	OP	Total
Archaea (A)	48	0	1	49
Bacteria (B)	239	72	110	421
Eukarya (E)	133	22	20	175
Total	420	94	131	645

Figure S2. **A phylogenomic species tree generated from the entire dataset of proteomes.** The phylogram of the most parsimonious rooted tree (tree length = 15,597 steps; CI (consistency index) = 0.083; RI (retention index) = 0.771; g_1 (tree skewness) = -0.288) describes the evolution of 645 proteomes and was generated from genomic abundances of 2,493 FFs (2,352 of which represent parsimony informative sites). Terminal nodes of Archaea (A: 49 proteomes), Bacteria (B: 421), and Eukarya (E: 175) were labeled in red, blue, and gray, respectively. The dotted lines explicitly display the borders between two superkingdoms. The life-styles of proteomes were displayed using a vertical bar beside their terminal leaves. Proteomes from free-living (420 proteomes), facultative parasitic (94), and obligate parasitic (131) organisms were labeled in blue, red, and cyan, respectively.

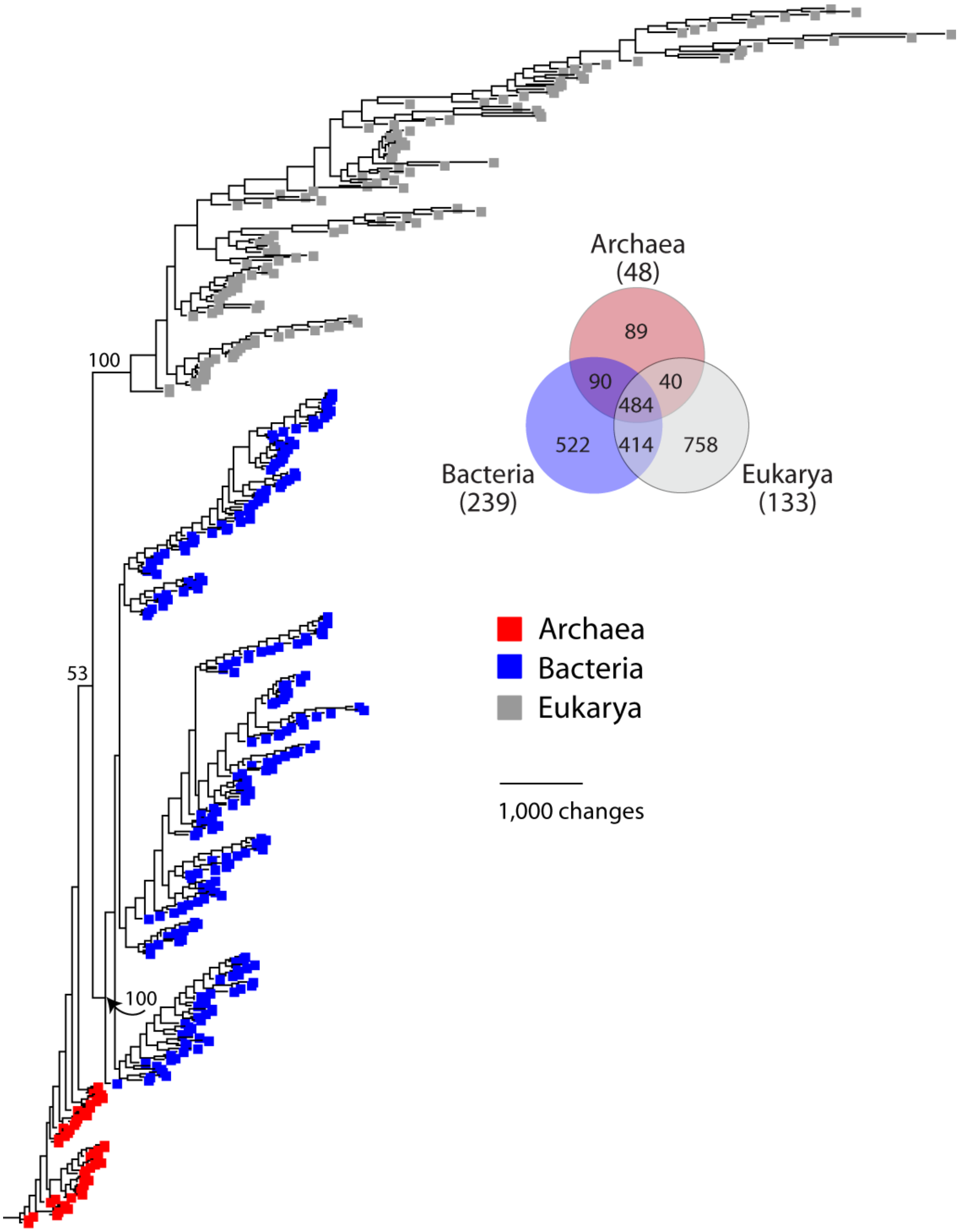

Figure S3. **A phylogenomic tree of proteomes describing the evolution of free-living organisms.** A most parsimonious rooted tree (tree length = 128,371 steps; CI = 0.103; RI = 0.760; g_1 = -0.241) was reconstructed from the genomic abundances of 2,397 FFs in 420 proteomes (2,262 of which represent parsimony informative sites). Terminal nodes of Archaea (A: 48 proteomes), Bacteria (B: 239 proteomes), and Eukarya (E: 133 proteomes) were labeled in red, blue, and gray, respectively. BS values >50% are shown above or below branches that cluster superkingdoms much higher groups. A Venn diagram shows the diversity of FFs in the three superkingdoms.

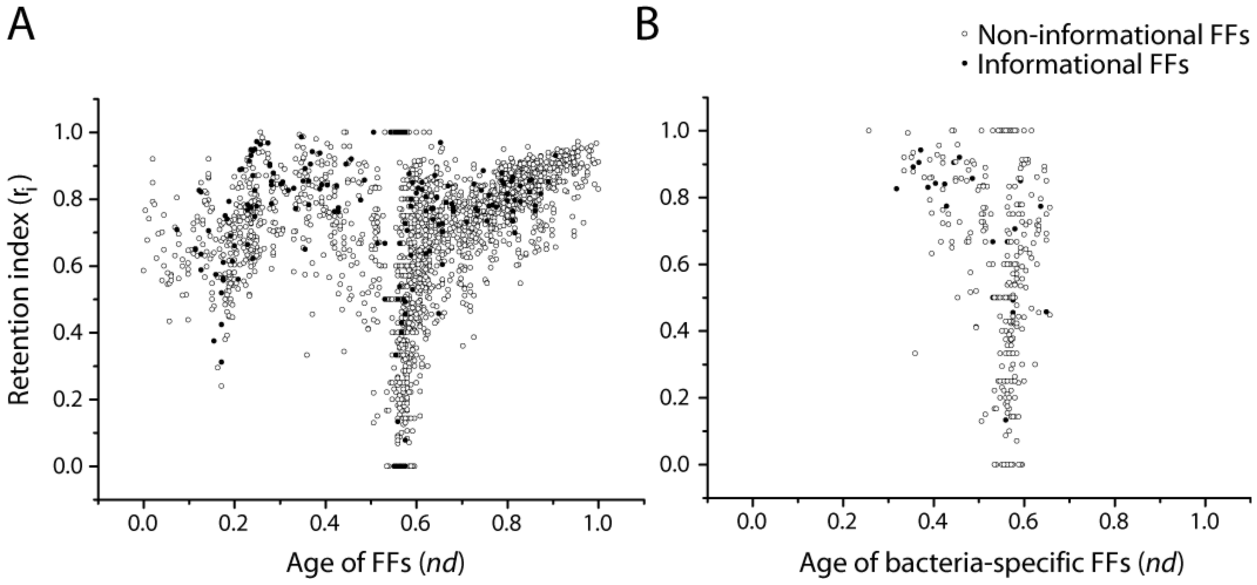

Figure S4. **The extent of homoplasy exhibited by phylogenomic characters (FFs) in trees of proteomes.** The retention index (r_i) of the FFs was determined from the tree of FL proteomes described in Figure 4. r_i values for the 2,262 parsimony-informative FFs (A) and 522 bacteria-specific FFs (B) were plotted against *nd* values obtained from a tree of FFs that is described in Figure 2A. A total of 182 and 34 out of the 2,262 and 522 FFs, respectively, were involved in informational cellular processes such as transcription, translation, and DNA replication (closed symbols).
